# Effects of Light Quality on Anthocyanin Biosynthesis and Related Gene Expression in *Camellia sinensis* ‘Ziyan’

**DOI:** 10.3390/ijms262210860

**Published:** 2025-11-09

**Authors:** Wei Li, Xiaoqin Tan, Jiacheng Huang, Wei Chen, Liqiang Tan, Qian Tang

**Affiliations:** 1 Faculty of Agriculture, Forestry and Food Engineering, Yibin University, Yibin 644005, China; lw816816@163.com; 2 College of Horticulture, Sichuan Agricultural University, Chengdu 611130, China; xqintan@163.com (X.T.); sicau_hjc@163.com (J.H.); chenwei2551@163.com (W.C.); tlq615@163.com (L.T.); 3 Tea Resources Utilization and Quality Testing Key Laboratory of Sichuan Province, Chengdu 611130, China

**Keywords:** blue light, tea plant, enzyme, anthocyanin biosynthesis, transcriptome

## Abstract

The purple-leaf tea cultivar ‘Ziyan’ is characterized by its high anthocyanin levels, which confer unique visual traits and health benefits. However, the effects of light quality on anthocyanin production remain poorly understood. This study explored the effects of red and blue light on anthocyanin biosynthesis in ‘Ziyan’, with white light as the control, using transcriptomic analysis, enzyme assays, and anthocyanin content measurements. The results showed that anthocyanin content increased under blue and red light, with blue light being the most effective, as the total anthocyanin content reached 81.79 mg/100 g FW, a 29.64% increase compared with white light. Delphinidin, cyanidin, and pelargonidin increased by 27.52%, 42.58%, and 102.72%, respectively. Transcriptome analysis showed red and blue light influenced photoreceptors and light signaling components, with decreased *COP*1 and increased *SPA*1 expression. Blue light upregulated key anthocyanin structural genes despite downregulating their transcription factors; it enhanced CHS, F3′H, F3′5′H, and ANS activities but decreased LAR and ANR activities, similar to the effect of red light. This research showed that the underlying mechanism may be achieved by coordinating light perception, gene expression, and enzyme activity. This study provides a theoretical basis for optimizing the light quality in purple tea plant cultivation.

## 1. Introduction

Tea plants (*Camellia sinensis*) are crops of considerable economic significance. Its new shoots are used to make tea beverages, which are one of the three most popular non-alcoholic drinks. Tea contains hundreds of secondary metabolites that confer various health benefits to the consumers. It contains approximately 4000 bioactive compounds, with polyphenols being the most prevalent [1,2]. Numerous studies have demonstrated that tea beverages contribute to the prevention of cancer and diabetes, assist in the regulation of blood pressure, suppress hyperglycemia, decrease reactive oxygen species, and reduce the risk of cardiovascular diseases [3,4,5,6,7,8].

Anthocyanins are naturally occurring plant pigments primarily located in the vacuoles of various plant organs. These compounds are responsible for the purple, blue, and red pigmentation observed in leaves, flowers, and fruits [9]. Extensive evidence has shown that anthocyanins play vital roles in mitigating various abiotic and biotic stresses, including ultraviolet damage, drought, and cold conditions [10,11,12]. Typically, those derived from green bud and leaf cultivars exhibits low anthocyanin concentrations. Nevertheless, there has been an increasing selection of anthocyanin-rich tea cultivars in recent years. For example, cultivars such as ‘Sunrouge’ [13], ‘Zijuan’ [14], and ‘Ziyan’ [15] are known to contain high levels of anthocyanins. The buds, leaves, and stems of the new shoots displayed a purple or red coloration. Furthermore, teas with elevated anthocyanin levels provide enhanced pharmacological benefits compared with conventional teas. For example, research has shown that anthocyanins in tea can increase the antioxidant capacity of the brain in murine models [16].

As is widely recognized, anthocyanins, a crucial class of flavonoids, are produced through the flavonoid biosynthetic pathway. This pathway involves several structural genes, including chalcone synthase (CHS), flavonoid 3′,5′-hydroxylase (F3′5′H), and anthocyanidin synthase (ANS). These genes are regulated by various transcription factors (TFs), including MYB, bHLH, and WD-repeat proteins, as well as the MBW complex formed by these factors [17,18]. Moreover, MYB repressors involved in anthocyanin biosynthesis play a significant role in the regulation of anthocyanin accumulation [6,19]. Furthermore, WRKY and NAC transcription factors have been implicated in the transcriptional control of anthocyanin synthesis [20,21,22,23].

Light, an environmental factor, including light quality, intensity, and photoperiod, markedly influences anthocyanin accumulation in plants. Red and blue light have been found to be advantageous for anthocyanin accumulation in apples and strawberries [24,25]. The effect of ultraviolet light on anthocyanin biosynthesis in ‘Ziyan’ is known, while the specific roles of red and blue light remain unclear. This study applied precise red and blue light-emitting diode treatments to ‘Ziyan’ to address this gap. We aimed to (1) quantify changes in anthocyanin and anthocyanidin contents, (2) analyze transcriptomic responses of light signaling and anthocyanin pathway genes, and (3) correlate these findings with key enzyme activities. This multi-level approach clarifies how distinct light qualities regulate anthocyanin production, providing insights into the optimization of light conditions for purple tea plant cultivation.

## 2. Results

### 2.1. Phenotypic Evaluation of the Second-Leaf Color Profile

**Figure 1:**
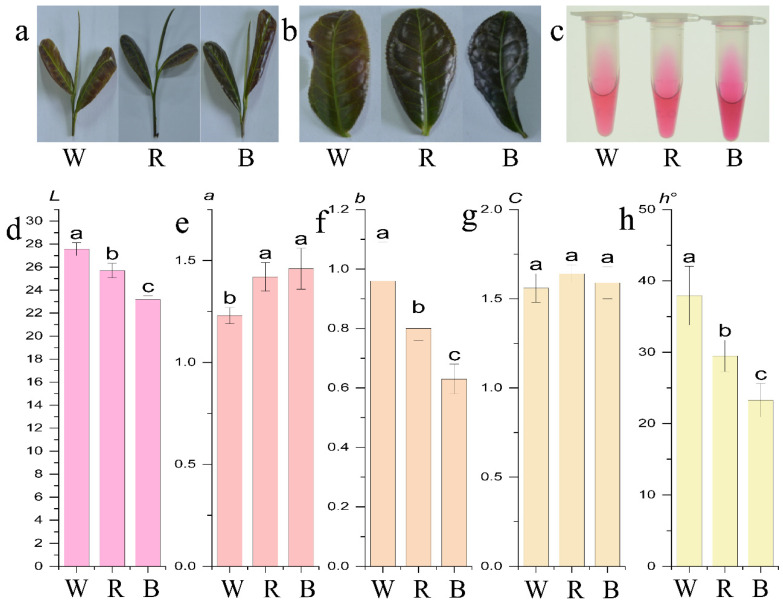
New shoot phenotype and anthocyanin extract of the new shoot of ‘Ziyan’ under different light quality treatments. (**a**) New shoot under different treatments; (**b**) the second top leaf; (**c**) anthocyanin extract solution; (**d**–**h**) the *L*, *a*, *b*, *C*, and *h*° values in the CIELAB color space. Different lowercase letters on the error bars represent significant differences (*p* < 0.05). The treatments with white, red, and blue lights are denoted as W, R, and B, respectively.

The color of the second leaf of one-bud-two-leaves (1B2L) was measured using a colorimeter and expressed as the *L a b* color space value. In the white light treatment, the values of *L*, *b*, and *h*° were higher than those in other treatments. In other words, the leaf color was brighter and yellower. The darker purple color of the leaves under blue light treatment was associated with the lowest *L*, *b*, and *h*° values and the highest a value. Based on the measurements, the leaf colors from deepest to lightest were ranked as follows: blue, red, and white light treatments (Figure 1).

### 2.2. Anthocyanin Content of ‘Ziyan’ New Shoots Under Different Light Quality

**Figure 2:**
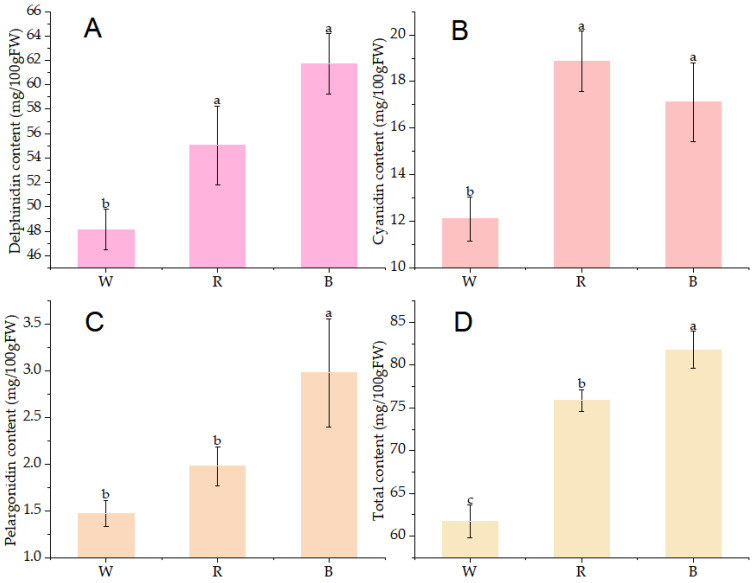
Anthocyanin content under different light qualities. (**A**–**D**) Delphinidin, cyanidin, pelargonidin, and total content. Different lowercase letters on the error bars represent significant differences (*p* < 0.05); The treatments with white, red, and blue light are denoted as W, R, and B, respectively.

High-performance liquid chromatography (HPLC) was employed to determine the anthocyanin profiles of the extracts from all experimental groups. A marked increase in total anthocyanin content was observed in the two light quality treatments compared to the white treatment (*p* < 0.05, Figure 2). The anthocyanin content reached a maximum of 81.79 mg/100 g FW under blue light, corresponding to a 29.64% increase compared with that in the control. The three predominant anthocyanidins, delphinidin, cyanidin, and pelargonidin were identified in all samples (Figure 2). Aside from cyanidin, the quantities of these two pigments in the blue light treatment exceeded those in other treatments. Compared to the control, the contents of these three pigments in new shoots under red and blue light treatments showed significant increases. Specifically, the levels of delphinidin, cyanidin, and pelargonidin increased by 27.52%, 42.58%, and 102.72%, respectively, in samples exposed to blue light compared with those in the control. These findings suggest that red and blue light promotes the accumulation of anthocyanins (including total and individual components) in ‘Ziyan’, with blue light exerting a particularly notable effect.

### 2.3. Catechins Content in New Shoots

We also measured catechin content in tea shoots. HPLC analysis revealed that both red and blue light treatments significantly enhanced the total and individual catechin contents compared with the control (Table 1). Blue light treatment elicited the most substantial enhancement, increasing the concentrations of gallocatechin (GC), catechin (C), epigallocatechin gallate (EGCG), epicatechin gallate (ECG), catechin gallate (CG), and total catechins by 4.00%, 57.14%, 104.05%, 104.05%, 120%, and 23.63%, respectively. These findings indicate that red and blue light promotes catechin accumulation, with blue light being particularly effective.

### 2.4. Analysis of Critical Enzymatic Activities in the Anthocyanin Synthetic Pathway

**Figure 3:**
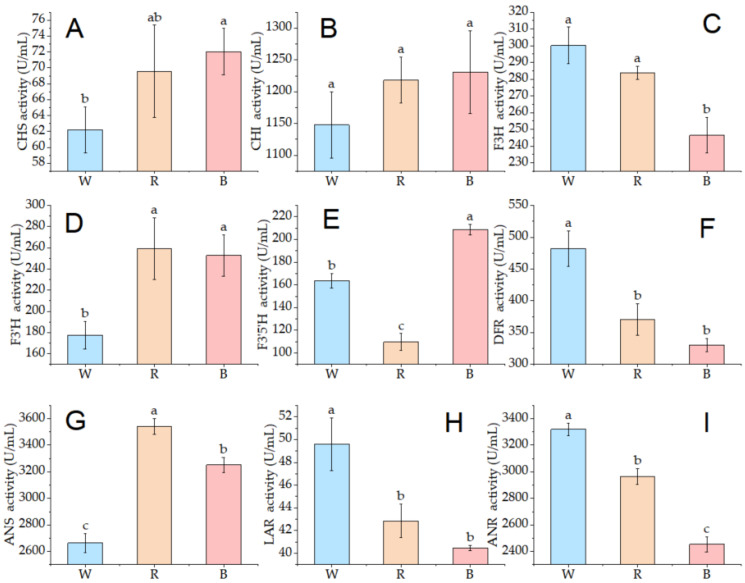
Activities of key enzymes involved in anthocyanin biosynthesis. Enzymatic activities shown are: (**A**) CHS, chalcone synthase; (**B**) CHI, chalcone isomerase; (**C**) F3H, flavone 3-hydroxylase; (**D**) F3′H, flavonoid 3′-hydroxylase; (**E**) F3′5′H, flavonoid 3′,5′-hydroxylase; (**F**) DFR, dihydroflavonol 4-reductase; (**G**) LAR, leucoanthocyanidin reductase; (**H**) ANS, anthocyanidin synthase; (**I**) ANR, anthocyanidin reductase. Different lowercase letters on the error bars represent significant differences (*p* < 0.05); The treatments with white, red, and blue light are denoted as W, R, and B, respectively.

The present investigation demonstrated that light quality significantly modulated the activities of the key enzymes involved in anthocyanin biosynthesis (Figure 3). Among them, CHS activity was most pronounced under blue light, showing a 15.84% increase over the white light control (*p* < 0.05), with no significant difference observed between red and blue light treatments (Figure 3A). However, CHI activity remained stable and was not significantly influenced by any light treatment (Figure 3B). F3H activity under both white and red light was notably higher than that under blue light, although no difference was detected between the former two (Figure 3C). Meanwhile, F3′H activity was significantly increased (>42.33%) under both colored light treatments compared to the control, with no significant difference between red and blue lights (Figure 3D). Compared to white light, blue light exposure increased F3′5′H activity by 27.46%, whereas red light significantly reduced it by 32.89% (Figure 3E, *p* < 0.05). The highest DFR activity was recorded under white light irradiation among all treatments (Figure 3F).

Nevertheless, no significant difference was observed between red and blue light treatments. Relative to white light, ANS activity increased significantly by 22.14% and 32.98% under red and blue light radiation, respectively (Figure 3G). The trend in LAR activity was similar to that of DFR (Figure 3H). ANR activity markedly decreased under red and blue light treatments compared to the control (Figure 3I). These findings suggest that red and blue light enhance the enzymatic activity associated with anthocyanin biosynthesis while decreasing the activity of ANR, which catalyzes the reduction of anthocyanidin.

### 2.5. Transcriptomic Profiling

Transcriptome sequencing was performed to investigate the molecular basis of anthocyanin biosynthesis in tea shoots under different light conditions. Sequencing of six cDNA libraries (W1, W2, R1, R2, B1, and B2) on the Illumina HiSeq 2500 platform generated approximately 56.05 Gb of clean data (187 million reads), with Q30 scores > 92.81% and GC content > 44.61% (Table S1). The alignment efficiency of these reads to the reference genome ranged from 67.85% to 68.15%. 

### 2.6. Differential Gene Expression Profiling

**Figure 4:**
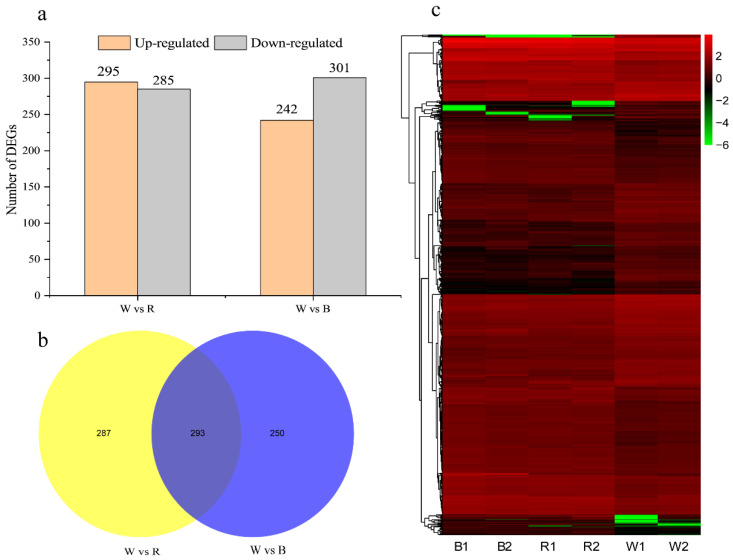
Analysis of different gene expression in new shoots of tea plants under different light qualities. (**a**) Number of DEGs in paired comparison of red and blue compared with white light; (**b**) Venn diagram of DEGs distribution; (**c**) hierarchical clustering diagram of DEGs. A color gradient from green to red depicts the expression levels of the DEGs. The color gradient from green to red indicates low to high expression levels. The labels W vs. R and W vs. B correspond to the gene sets differentially expressed between white light versus red light and white light versus blue light, respectively. B1, B2, R1, R2, W1, and W2 represent the two biological replicates.

Comparative analysis between white and red light treatments revealed 580 DEGs (295 upregulated and 285 downregulated under red light), whereas the W vs. B comparison identified 543 DEGs (242 upregulated and 301 downregulated under blue light) (Figure 4a). Of these, 293 DEGs were common to both comparisons, whereas 287 and 250 DEGs were specifically identified as unique to the W vs. R and W vs. B comparisons, respectively (Figure 4b). Cluster analysis of the DEGs under the three light treatments revealed that the samples exposed to red and blue light clustered together, indicating similar gene expression profiles (Figure 4c). These findings suggest that red and blue lights exert comparable effects on the regulation of gene expression.

### 2.7. Functional Enrichment Analysis of DEGs

**Figure 5:**
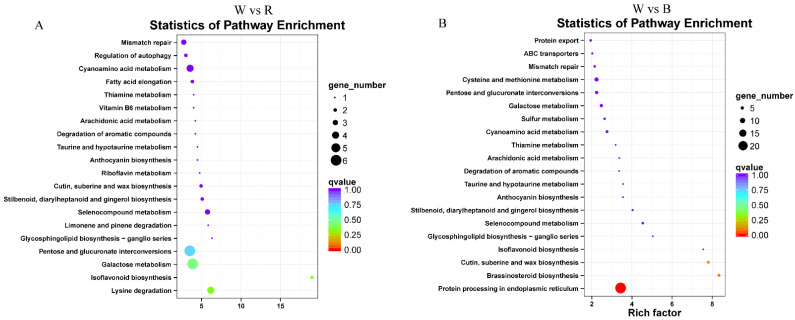
Significantly enriched KEGG pathways identified from DEGs in pairwise comparisons of (**A**) W vs. R and (**B**) W vs. B. Pathways are displayed on the *Y*-axis against the enrichment factor (*X*-axis). The dot size scales with the DEG count, and the color represents the significance level.

Gene Ontology (GO) and KEGG pathway analyses were employed to interpret the functions of the identified DEGs. GO analysis categorized the DEGs into three functional domains (Figure S1). The most significantly enriched terms were “metabolic process,” “cell part,” and “catalytic activity” for biological process, cellular component, and molecular function, respectively, with highly similar results for both W vs. R and W vs. B comparisons. KEGG enrichment analysis revealed distinct pathway associations for the different light quality comparisons. For the W vs. R comparison, while no pathways met the threshold for statistical significance, the DEGs were primarily associated with ‘Pentose and glucuronate interconversions’, ‘Lysine degradation’ and ‘Galactose metabolism’ based on their ranking in the enrichment analysis. In contrast, for the W vs. B comparison, DEGs were significantly enriched in ‘Protein processing in the endoplasmic reticulum’ (Figure 5). These findings suggest that red and blue light may influence the metabolism of tea shoots through distinct molecular mechanisms.

### 2.8. Analysis of Light Signal Perception and Transduction

**Figure 6:**
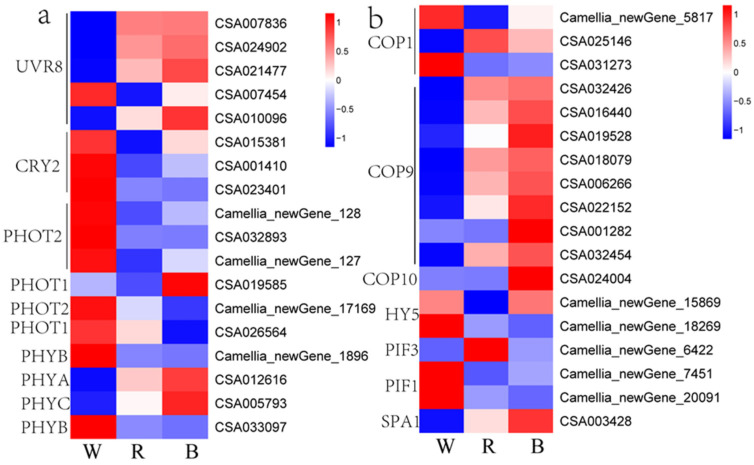
Heatmap of gene expression for (**a**) photoreceptors and (**b**) photosignal transduction components across light treatments.

Gene function annotation identified transcripts encoding four major photoreceptor families: phytochromes (PHYs), phototropins (PHOTs), cryptochromes (CRYs), and ultraviolet (UV) RESISTANCE LOCUS 8 (UVR8). Expression analysis revealed that transcript levels of most of these photoreceptor genes were downregulated under both red and blue light treatments compared to the white light control (Figure 6a). The repressors of light signal transduction, *COP1*, *COP9*, and *COP10*, exhibited varying expression levels under white, red, and blue light irradiation. For the *COP* family, the expression levels of *COP9* and *COP10* increased, whereas the transcript level of *COP1* decreased under red and blue light treatments. *SPA1* showed the highest expression under blue light treatment. Within the *PIF* family, *PIF1* and *PIF3* were induced by white and red light, respectively. Notably, red and white light exerted opposing effects on the expression of *HY5*, which encodes a positive regulator of photomorphogenesis, with significant suppression and promotion, respectively (Figure 6b). These findings demonstrate that different light qualities significantly influence the induction of photoreceptors and light signal transduction components in tea plants. Coupled with the notable differences in anthocyanin levels under varying light conditions, we hypothesize that this may be related to the interaction between photoreceptors and light signal transduction components, thereby regulating anthocyanin biosynthesis in tea shoots.

### 2.9. Transcription Factor Profiling

**Figure 7:**
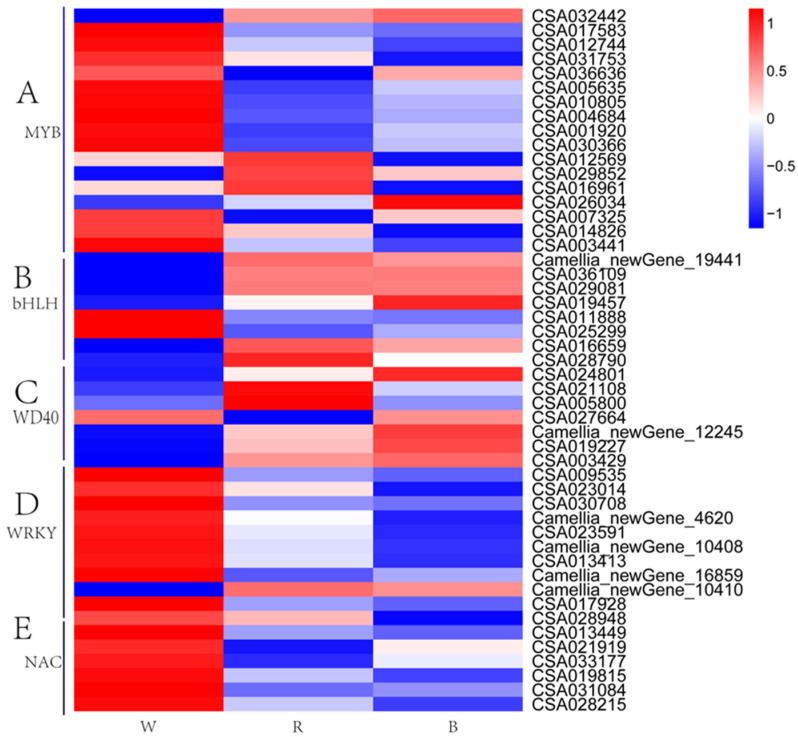
Transcription factor expression patterns under different light quality treatment. (**A**) MYB; (**B**) bHLH; (**C**) WD40; (**D**) WRKY; (**E**) NAC.

A total of 59 TFs, annotated as 27 *MYB*s (Figure 7A), 8 *bHLH*s (Figure 7B), 7 *WD40*s (Figure 7C), 11 *WRKY*s (Figure 7D), and 6 *NAC*s (Figure 7E), were identified as potential participants in the regulation of flavonoids and anthocyanins (Figure 7). Most of these gene expression patterns were consistent under R and B treatments. Only one *MYB* gene (CSA032442) was significantly upregulated, whereas three (CSA017583, CSA012744, and CSA030366) were downregulated in the W vs. B group. In addition, five *MYB*s (CSA017583, CSA005635, CSA010805, CSA004684, and CSA001920) showed decreased expression in the W vs. R group. Regarding the *bHLH* family, most genes were repressed under white light; however, they were notably upregulated in the B treatment, including Camellia_newGene_19441, CSA036109, CSA029081, and CSA019457. Compared with the white light control, nearly all *WD40* genes displayed higher expression levels under both red and blue light treatments. Furthermore, the expression patterns of the *WRKY* and *NAC* transcription factor families indicate their participation in the complex regulatory network governing anthocyanin biosynthesis. Although tea shoots exhibited high anthocyanin concentrations following red and blue light treatments, most *WRKY* genes were downregulated. These findings suggest that the aforementioned transcription factors may play crucial roles in modulating anthocyanin synthesis in purple-leaf tea plants exposed to varying light qualities.

### 2.10. DEGs in the Anthocyanin Biosynthesis Pathway

**Figure 8:**
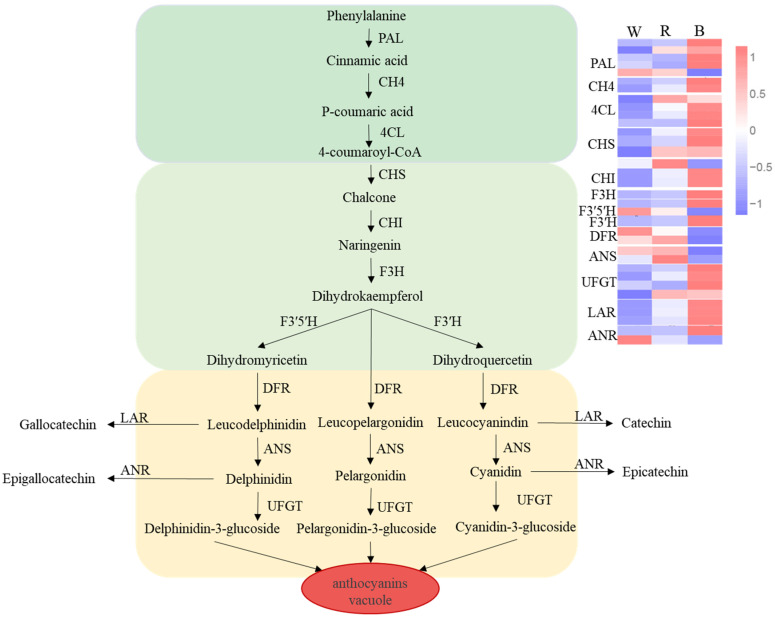
Expression of anthocyanin biosynthetic genes. The color scale on the right represents the normalized value of log10 (FPKM) and FPKM (fragments per kilobase of transcript per million mapped reads) values obtained from RNA-seq data. The expression levels of each gene are shown by colors ranging from low (blue) to high (red).

The analysis of DEGs from pairwise comparisons focused on 36 transcripts representing 14 key anthocyanin biosynthetic genes, which predominantly belonged to multi-gene families (with the exception of *F3H* and *F3′H*). In the W vs. B group, genes involved in phenylpropanoid and early biosynthetic steps were upregulated. However, one *CHI* was strongly induced specifically by red light, as were the late-stage genes *DFR* and *ANS*, which were suppressed by blue light (Figure 8). Compared to the white light control, *UFGT*, the final gene involved in anthocyanin biosynthesis, was upregulated under blue light treatment, which was consistent with the increase in anthocyanin content. Additionally, elevated *FLS*, *LAR*, and *ANR* levels under red and blue light treatments likely contributed to catechin accumulation. Overall, these results suggest that blue and red light more effectively induce anthocyanin accumulation in ‘Ziyan’ leaves than does white light.

### 2.11. qRT-PCR Validation of Gene Expression

The expression patterns of the fifteen selected genes, as analyzed by quantitative real-time PCR (qRT-PCR), were highly consistent with the RNA-seq data. The analysis revealed a strong positive correlation (r = 0.804, *p* < 0.001) between the two datasets, effectively demonstrating the high reproducibility and reliability of our transcriptomic findings (Figure S2).

## 3. Discussion

Plant genetic characteristics are fundamental to anthocyanin biosynthesis; however, environmental factors, both biotic and abiotic, also substantially influence the qualitative and quantitative levels of these compounds [26]. This study used high-performance liquid chromatography (HPLC) to measure anthocyanin levels in new shoots of purple-leaf tea plants, aiming to investigate how red and blue light influence anthocyanin synthesis. The results demonstrated that both blue and red light markedly increased anthocyanin content in the purple tea plant ‘Ziyan’ (Figure 2). Blue light treatment produced the highest anthocyanin concentrations, followed closely by red light. This pattern is consistent with the results documented in other plant species, such as grapes, bilberries, and strawberries [25,27,28]. Previous studies have indicated that varying light qualities induce different responses in anthocyanin biosynthesis [29].

In most cases, short-wavelength light, such as ultraviolet and blue light, promotes greater growth [30,31,32]. Similarly, evidence indicates that long-wavelength light can boost anthocyanin content in plants [33]. This study found that delphinidin, cyanidin, pelargonidin, and total anthocyanins increased by 28.19%, 41.17%, 102.72%, and 32.52%, respectively, under blue light treatment compared to that in the control. It is clear that blue light significantly affects the pigment content in pelargonium, demonstrating that light quality influences the levels of different anthocyanin components and can change their proportions. This is further supported by the varying proportions of anthocyanins in strawberries subjected to different light quality treatments [25].

Plants possess multiple photoreceptors capable of detecting various wavelengths of light, thereby initiating a cascade of signal transduction events that facilitate diverse physiological and biochemical responses [34]. This study employed transcriptome analysis to investigate the genetic mechanisms by which monochromatic red and blue light affects anthocyanin accumulation in tea shoots. Compared with white light treatment, the expression levels of the photoreceptors *CYR*2, *PHOT*2, and *PHYB* were reduced under red and blue light. Conversely, most UVR8 receptor genes demonstrated the opposite trend. It has been reported that CRY photoreceptors typically carry out the synthesis of anthocyanins in plants in response to blue light [35,36], and this regulation often requires PHY activity for greater effectiveness [37,38,39]. Furthermore, the interaction between CRY and UVR8 is essential for modulating the expression of target genes under natural conditions [40,41]. The study revealed that anthocyanin levels were markedly elevated under blue light conditions compared to red and white light, even though the expression pattern of CYR receptors declined. This observation implies that anthocyanin synthesis is likely governed by the interplay between various receptors. Furthermore, red and blue light treatments stimulated the expression of SPA1 but suppressed the expression of the negative regulator COP1. In apples, light-induced anthocyanin biosynthesis is orchestrated by the interaction between MdCOP1 ubiquitin E3 ligases and the MdMYB1 transcription factor [42]. Similarly, in *Arabidopsis thaliana*, the COP1/SPA complex modulates the stability of the MYB transcription factors PAP1 and PAP2 via the ubiquitin-proteasome pathway, thereby governing anthocyanin accumulation [43], revealing a conserved mechanistic basis for the light-mediated regulation.

In the realm of light-induced anthocyanin synthesis, the MYB transcription factor is integral to the regulation of structural gene expression and subsequent anthocyanin accumulation. For example, under shaded conditions, the transcription of LrMYB15 in lily ceases entirely, as does the coloration of the flower, indicating that the transcriptional activity of this gene is light-dependent and that light modulates the expression level of MYB to regulate anthocyanin accumulation [44,45]. Conversely, the expression levels of most MYBs identified in this study decreased under both red and blue light treatments, which was inconsistent with the observed quantitative results for anthocyanins. A possible explanation for this discrepancy is that the MYBs identified may not be directly involved in anthocyanin biosynthesis in tea shoots, or they may function as inhibitory, regulatory transcription factors. Similarly, the expression patterns of *WRKY* and *NAC* gene family members detected in this study remained low in response to red and blue light treatments, whereas most *bHLH* and *WD40* transcription factor family genes showed higher expression levels compared to white light conditions. Based on the elevated anthocyanin levels observed in both treatments, we hypothesized that bHLH and WD40 might participate in the regulation of anthocyanin biosynthesis in *Camellia sinensis*. Expression profiling of anthocyanin biosynthetic genes under blue light showed divergent regulation between the early and late stages. Early genes were highly expressed, but the transcript levels of late genes (e.g., *DFR* and *ANS*) were suppressed, a trend corroborated by enzyme activity measurements. Interestingly, this general transcriptional suppression was inversely correlated with the peak anthocyanin content observed under blue light. This apparent paradox may be explained by a potential feedback mechanism in which a high anthocyanin concentration leads to the downregulation of biosynthetic genes. This hypothesis is consistent with our previous observations in ‘Ziyan’ tea shoots, where the highest anthocyanin content was detected at the earliest developmental stage (one bud with two leaves) and decreased as the shoots matured (to one bud with six leaves), paralleled by a reduction in gene expression. The above explanation may also account for the inconsistency observed between the enzyme activity and expression patterns of ANS. When anthocyanin levels reach saturation, negative feedback mechanisms may inhibit gene expression, indirectly indicating that blue light promotes rapid anthocyanin accumulation in tea shoots. Additionally, this study revealed that *UFGT* expression was highest under blue light, favoring the formation of stable anthocyanins.

Catechin synthesis occurs through competition with anthocyanins for shared substrates or via direct reduction of anthocyanins. To gain a more comprehensive understanding of how light quality, specifically red and blue light, stimulates anthocyanin production, we investigated the catechin content. This study revealed an apparent discrepancy between the reduced activities of LAR and ANR under blue and red light and the concurrent increase in catechin levels. Furthermore, the transcript levels of *LAR* and *ANR* were upregulated under these treatments. These findings indicate a metabolic flux shift toward catechin synthesis rather than anthocyanin synthesis during the subsequent development of tea shoots. Consequently, red and blue light not only effectively enhanced the anthocyanin content in purple foliage tea but also increased catechin levels, as corroborated by a previous study [46].

## 4. Materials and Methods

### 4.1. Tea Plant Cultivation and Light Treatments

One-year-old ‘Ziyan’ was used in this study. Healthy, uniform plants were transplanted into plastic pots (45 cm × 30 cm × 25 cm), with 36 plants per pot, and then pruned to a height of 25 cm. Tea plants were grown in a controlled-environment chamber (Shanghai Sanfa Technology Co., Ltd., Shanghai, China) under a 14/10 h (light/dark) photoperiod, with temperatures of 25/18 °C, and relative humidity of approximately 80%. The experimental treatments comprised white light (W, control), red light (R), and blue light (B), all at a photon flux density of 200 µmol m^−2^ s^−1^. Sampling was conducted when the majority of young shoots reached the one-bud-two-leaves (1B2L) stage. The harvested samples (1B2L) were immediately frozen in liquid nitrogen and maintained at −80 °C. The samples were designated for subsequent analysis of RNA, pigments, catechins, and enzyme activity. The experiment included two biological replicates for RNA-seq and three for all other measurements (pigments, catechins, enzyme activity, and qRT-PCR).

### 4.2. Measurement of Color Parameters

The color of the second top leaf from the samples was quantified using a CM-2600d spectrophotometer (Konica Minolta, Tokyo, Japan) with a D65 illuminant and 45°/normal geometry. Each leaf was measured five times, avoiding the midrib. Color was recorded in the *L a b* color space, where *L* represents lightness, *a* indicates the green (−) to red (+) spectrum, and *b* represents the blue (−) to yellow (+) spectrum. The chroma (*C*) and hue angle (*h*°) were also calculated.

### 4.3. Quantification of Anthocyanins by HPLC

Anthocyanins were extracted from fresh leaves that were frozen in liquid nitrogen and ground to a fine powder. About 0.2 g of powder was homogenized with 4 mL of methanol containing 1% (*v*/*v*) hydrochloric acid and incubated at 4 °C for 2 h. The supernatant was collected after centrifugation (5000 rpm, 10 min). The residue was subsequently re-extracted twice using 3 mL of the extraction solvent for 2 h and 12 h. The combined supernatants were subjected to acid hydrolysis for HPLC analysis [47]. Briefly, the extract (0.2 mL) was combined with 0.4 mL of 5 mol·L^−1^ HCl, heated at 90 °C for 30 min for hydrolysis, and then immediately cooled on ice. Analysis was performed on an Agilent 1260 HPLC system equipped (Palo Alto, CA, USA) with a VWD detector and a Titank C18 column (Torrance, CA, USA). The mobile phase consisted of (A) water:acetonitrile:formic acid (87:3:10, *v*/*v*/*v*) and (B) 100% acetonitrile, using a gradient from 15% to 30% B over 30 min. Chromatographic separation was achieved under the following conditions: flow rate, 1.0 mL·min^−1^; injection volume, 10 μL; column temperature, 35 °C; detection wavelength, 520 nm. Anthocyanidins were quantified using external standards (peonidin, pelargonidin, delphinidin, malvidin, and cyanidin chlorides; ChromaDex, Los Angeles, CA, USA).

### 4.4. Enzyme Activity Assays

The activities of the key enzymes involved in anthocyanin biosynthesis were quantified using commercial plant ELISA kits (Shanghai Fusheng Industry Co., Ltd., Shanghai, China). Briefly, 0.5 g of frozen tissue was homogenized in liquid nitrogen and mixed with 4.5 mL of phosphate-buffered saline (PBS, 0.01 mol/L, pH 7.4). The homogenate was centrifuged at 5000 rpm for 15 min at 4 °C, and the resulting supernatant was collected for further analysis. ELISA was performed according to the manufacturer’s instructions. After a 20-min equilibrium of all reagents at room temperature, 10 μL supernatant was combined with 40 μL sample diluent in a microplate well. Then, 100 μL of horseradish peroxidase (HRP)-conjugated reagent was added to each well. The plates were sealed and incubated at 37 °C for 60 min. After incubation, the liquid was discarded, and the wells were washed five times with 400 μL of wash buffer. Subsequently, 50 μL each of chromogen solutions A and B were added, followed by a 15-min incubation at 37 °C in the dark. The reaction was stopped with 50 μL stop solution, and the absorbance was measured at 450 nm within 15 min.

### 4.5. Quantification of Catechins by HPLC

Catechin was extracted from the samples according to the Chinese National Standard (GB/T8313-2018) [48]. HPLC analysis was conducted on an Agilent 1260 system equipped with a Phenomenex Titank C18 column. The mobile phases were (A) acetonitrile/acetic acid/EDTA (90:20:0.02, *v*/*v*/*w*) and (B) acetonitrile/acetic acid/EDTA (800:20:0.02, *v*/*v*/*w*). The elution program was as follows: 100% A for 8 min, a linear gradient to 68% A over 15 min, followed by a 10-min hold. The injection volume was 10 μL, flow rate was 1 mL/min, column temperature was 35 °C, and the detection wavelength was 278 nm. Catechin standard samples were obtained from Sigma-Aldrich (Saint Louis, MO, USA).

### 4.6. Transcriptome Analysis by RNA-Sequencing

TRIzol Reagent (Invitrogen, Carlsbad, CA, USA) was used to extract Total RNA. RNA concentration, purity, and integrity were determined using a NanoDrop 2000 spectrophotometer (Thermo Fisher Scientific, Waltham, MA, USA), NanoPhotometer spectrophotometer (IMPLEN, Los Angeles, CA, USA), and Agilent Bioanalyzer 2100 system with the RNA Nano 6000 Assay Kit (Palo Alto, CA, USA), respectively. Sequencing libraries were constructed from 1.5 μg of total RNA per sample for six samples (two biological replicates per treatment: W, R, and B) using the NEBNext Ultra™ RNA Library Prep Kit for Illumina (NEB, MA, USA). Briefly, mRNA was enriched with poly-T magnetic beads and fragmented. First- and second-strand cDNA were synthesized, end-repaired, adenylated, and ligated to NEBNext adaptors. cDNA fragments of ~240 bp were size-selected using AMPure XP beads (Beckman Coulter, Brea, CA, USA). After USER enzyme digestion and PCR amplification, the final library quality was assessed on the Agilent Bioanalyzer. The libraries were clustered on a cBot Cluster Generation System and sequenced on an Illumina HiSeq 4000 (San Diego, CA, USA) platform to generate paired-end reads.

### 4.7. RNA-Seq Data Alignment and Quantification

To ensure the integrity of the sequencing data, low-quality reads were filtered out using a custom Perl script. This process removed sequences that were exclusively composed of adapter contaminants, contained over 5% of undetermined (unknown) nucleotides, or displayed a Q20 score lower than 20%, where Q20 represents the percentage of bases with a sequencing error rate under 1%. The retained high-quality reads were aligned to the *Camellia sinensis* ‘Yunkang10’ reference genome (available at https://tpia.teaplants.cn/download.html, 28 September 2025) using Tophat2 [49]. Following alignment, PCR duplicates were removed from the BAM files, and transcript abundances were estimated using Cufflinks. Gene expression levels were quantified using the FPKM value [50].

### 4.8. Differential Gene Expression Analysis

DEGs between the comparison groups (W vs. R and W vs. B) were identified using the DESeq2 package [51]. The significance of expression differences was determined by applying a false discovery rate (FDR) correction to the *p*-values from multiple tests. Genes with |log_2_(fold change)| ≥ 1 and an FDR < 0.01 were defined as DEGs for subsequent analyses.

### 4.9. Functional Enrichment Analysis of Degs

Functional enrichment analysis of DEGs was performed to interpret their biological significance. Gene Ontology (GO) term enrichment was analyzed using the GOseq R package (version 1.0.3) [52], which employs the Wallenius non-central hypergeomet-ric distribution to correct for gene length bias. The statistical enrichment of DEGs in KEGG pathways [53] was assessed using KOBAS software (version 1.0) [54].

### 4.10. qRT-PCR Validation

The reliability of the RNA-seq data was verified by qRT-PCR using the CFX96 system (Irvine, CA, USA). with three replicates. cDNA was synthesized from total RNA using a PrimeScript™ RT Kit with gDNA Eraser (Takara, Kyoto, Japan), followed by qPCR amplification with SYBR Premix (Takara, Kyoto, Japan) and gene-specific primers (Table S2). Data were normalized to *CsPTB* [55] and analyzed via the 2^−ΔΔCt^ method [56].

### 4.11. Statistical Analysis

Data are expressed as mean ± SD (*n* = 3) and analyzed using SPSS 20.0. Statistical significance was assessed by one-way ANOVA with Duncan’s test for multiple comparisons.

## 5. Conclusions

Blue and red light promoted anthocyanin accumulation in ‘Ziyan’, with blue light showing the strongest effect. Under blue light, delphinidin, cyanidin, pelargonidin, and total anthocyanins increased by 27.52%, 42.58%, 102.72%, and 29.64%, respectively, compared with white light treatment. Transcriptomic analysis showed that photoreceptor and signal transduction genes were distinctly modulated by different light qualities, whereas most transcription factor genes showed similar expression under red and blue light. Most structural genes involved in anthocyanin biosynthesis were upregulated under both light treatments. Blue light enhanced CHS, F3′H, F3′5′H, and ANS activities, while both lights suppressed LAR and ANR activities, with increased catechin content. These findings show that blue and red light can stimulate anthocyanin and catechin biosynthesis in purple tea cultivation.

## Figures and Tables

**Table 1:** Catechin content indifferent treatment (mg/g, DW).

Component	White Light	Red Light	Blue Light
GC	5.0 ± 0.1 b	4.8 ± 0.1 c	5.2 ± 0.1 a
EGC	34.4 ± 1.4 a	26.0 ± 0.8 b	25.2 ± 1.1 b
C	1.4 ± 0.0 c	1.6 ± 0.1 b	2.2 ± 0.1 a
EC	5.4 ± 0.3 a	3.3 ± 0.1 b	2.7 ± 0.2 c
EGCG	32.0 ± 0.8 c	44.4 ± 1.8 b	54.6 ± 0.7 a
GCG	1.0 ± 0.2 a	0.5 ± 0.1 b	1.1 ± 0.1 a
ECG	7.4 ± 0.1 c	11.6 ± 0.5 b	15.1 ± 0.2 a
CG	1.0 ± 0 c	1.4 ± 0.1 b	2.2 ± 0.1 a
Total	87.6 ± 1.9 c	93.8 ± 2.4 b	108.3 ± 1.1 a

Different letters within a row indicate significant differences (*p* < 0.05). GC: gallocatechin; EGC: epigallocatechin; C: catechin; EC: epicatechin; EGCG: epigallocatechin gallate; GCG: gallocatechin gallate; ECG: epicatechin gallate; CG: catechin gallate.

## Data Availability

The original contributions presented in this study are included in the article/Supplementary Materials.

## Supplementary Materials

The following supporting information can be downloaded at: https://www.mdpi.com/article/10.3390/ijms262210860/s1, Figure S1: Gene Ontology (GO) enrichment analysis in pairwise comparisons of (W vs R) and (W vs B); Figure S2: Validation of gene expression by qRT-PCR; Table S1: Summary of RNA-Sequencing output and read mapping statistics; Table S2: Primers used for qRT-PCR analysis.
